# Therapeutics of Alzheimer’s Disease: Recent Developments

**DOI:** 10.3390/antiox11122402

**Published:** 2022-12-03

**Authors:** Scott Burns, Ashley Selman, Ujala Sehar, Priyanka Rawat, Arubala P. Reddy, P. Hemachandra Reddy

**Affiliations:** 1 Department of Internal Medicine, Texas Tech University Health Sciences Center, Lubbock, TX 79430, USA; 2 Neurology, Departments of School of Medicine, Texas Tech University Health Sciences Center, Lubbock, TX 79430, USA; 3 Public Health Department of Graduate School of Biomedical Sciences, Texas Tech University Health Sciences Center, Lubbock, TX 79430, USA; 4 Department of Speech, Language and Hearing Sciences, School Health Professions, Texas Tech University Health Sciences Center, Lubbock, TX 79430, USA; 5 Nutritional Sciences Department, College of Human Sciences, Texas Tech University, Lubbock, TX 79409, USA

**Keywords:** Alzheimer’s disease, therapeutics, mitochondria, mitochondrial-targeted molecules, anti-amyloid beta, BAC1 inhibitors, monoclonal antibodies, anti-phosphorylated, anti-oligomeric, anti-inflammatory, hormonal, antioxidant, synaptic, anti-sleep

## Abstract

With increasing aging, dementia is a growing public health concern globally. Patients with dementia have multiple psychological and behavioral changes, including depression, anxiety, inappropriate behavior, paranoia, agitation, and hallucinations. The major types of dementia are Alzheimer’s disease (AD), vascular dementia (VCID), Lewy body dementia (LBD), frontotemporal dementia (FTD), and mixed dementia (MiAD). Among these, AD is the most common form of dementia in the elderly population. In the last three decades, tremendous progress has been made in understanding AD’s biology and disease progression, particularly its molecular basis, biomarker development, and drug discovery. Multiple cellular changes have been implicated in the progression of AD, including amyloid beta, phosphorylated tau, synaptic damage, mitochondrial dysfunction, deregulated microRNAs, inflammatory changes, hormonal deregulation, and others; based on these changes, therapeutic strategies have been developed, which are currently being tested in animal models and human clinical trials. The purpose of our article is to highlight recent therapeutic strategies’ developments, critically discuss current strategies’ failures, and propose new strategies to combat this devasting mental illness.

## 1. Introduction

**Figure 1:**
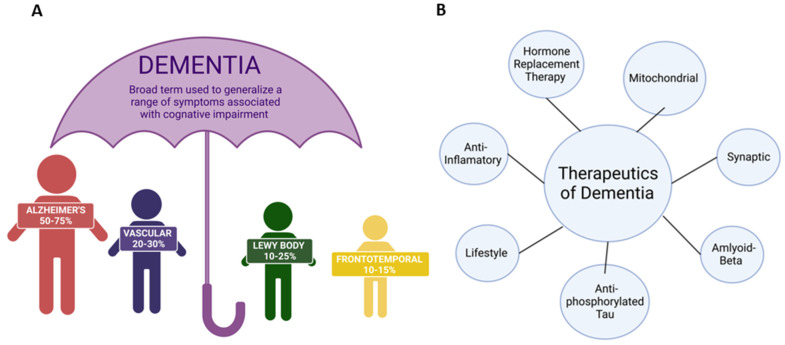
Types and therapeutics of dementia. (**A**) The significant types of dementia are Alzheimer’s disease, Lewy body dementia, Vascular dementia, and frontotemporal dementia. (**B**) Possible therapeutic strategies to treat dementia.

Dementia is a growing problem in the United States (U.S.). Dementia is “the acquired loss of cognition in multiple cognitive domains sufficiently severe to affect social or occupational function” [1]. According to the Mayo Clinic in Minnesota, these cognitive changes commonly occur with dementia: (1) memory loss that is usually noticed by someone else, (2) difficulty with communication or recalling words, (3) difficulty with visual and spatial ability, (e.g., getting lost while driving), (4) confusion and disorientation, (5) difficulty reasoning or problem-solving, (6) difficulty with complex tasks, (7) difficulty with planning and organization, and (8) difficulty with coordination and motor functions (Alzheimer’s Association, 2019) [2,3]. Dementia patients also commonly have psychological changes, such as a change in personality, depression, anxiety, inappropriate behavior, paranoia, agitation, and hallucinations [4]. The major types of dementia are Alzheimer’s disease (AD), vascular dementia, Lewy body dementia (LBD), frontotemporal dementia, and mixed dementia [5]. Figure 1 represents different types and treatment strategies for dementia. These types of dementia are irreversible and progressive. Dementia is often coupled with more than one neuropathology and is most associated with cerebrovascular pathology. Dementia yields several changes at the cellular level, such as synaptic loss, nonfunctional mitochondria, formation of amyloid-β plaques and active phosphorylated tau in the brain, reactive astrocytes, and the rapid division of microglia [6]. The pace at which symptoms of dementia advance from mild to moderate to severe differs from person to person. Studies indicate that women are more likely than men to get dementia due to biological differences, such as cognition, anatomy, longevity, societal norms, and opportunities in life [3,7].

The prevalence of dementia is expected to increase from around 57 million global cases in 2019 to about 152 million cases by 2050 [8]. Approximately 6 million people were affected by AD in the United States in 2019 [1]. AD is characterized by biomarker evidence of Alzheimer’s-related brain changes and noticeable symptoms involving memory, thinking, behavior, and language. As AD progresses, multiple types of symptoms change over time during the disease [9]. These symptoms suggest the degree of damage to neurons in different brain parts.

Vascular dementia is defined as dementia that damages the blood supply to the brain. These blood vessel abnormalities can cause numerous clinical complications (e.g., stroke) or ischemic brain damage to the fibers of the white matter in the brain. Most commonly, this dementia is associated with issues in problem-solving, slow thinking, and poor focus rather than memory loss [10]. Lewy body dementia (LBD) is defined as balloon-like clumps of protein lodged within the brains of those with AD and Parkinson’s disease (PD). Lewy body patients commonly experience visual hallucinations, tremors, and rigidity [11]. Frontotemporal dementia is characterized by the breakdown of nerve cells and associated connections in the temporal and frontal lobes of the brain [12]. As a result, these patients often experience greater changes to their language skills, personalities, thinking, and judgment. Finally, mixed dementia is the confluence of many different types of dementia co-occurring. This type of dementia is typically identified during postmortem autopsies. Current clinical research is underway to determine how mixed dementia presents in a constellation of different and variable symptoms.

A dementia diagnosis requires a physician to obtain a patient history to document cognitive decline and impairment in daily activities (with family and friends in agreement). A physician must also conduct an examination to identify a moderately impaired mental status, specifically impairments in memory, language, attention, and visual-spatial cognition (e.g., spatial orientation, executive function, mood) [1]. A dementia diagnosis also presents a significant economic and time burden for patients and their families. Family members often become the primary caretakers for those diagnosed with dementia. According to the Department of Texas Health and Human Services, family members and friends provided more than USD 271 billion in unpaid care to people living with AD and other types of dementia in 2021 (Texas Department of State Health Services, 2017).

Several traditional therapeutics can help slow disease progression and provide modest symptomatic relief for patients with dementia. Still, it cannot prevent or significantly improve the health of those already affected by severe dementia. For example, acetylcholinesterase inhibitors (e.g., donepezil) treat mild-to-severe AD, memantine (alone or as an add-on therapy) treats moderate-to-severe AD, and rivastigmine is an approved treatment for dementia associated with PD [1].

Therapeutics for the treatment of dementia is an expanding research field with new drugs and medicines in clinical animal trials. This review article discusses current and new therapeutics for the treatment of dementia: (1) mitochondrial therapeutics (e.g., mitochondrial-targeted molecules), (2) anti-amyloid beta therapeutics (e.g., BAC1 inhibitors, monoclonal antibodies), (3) anti-phosphorylated and anti-oligomeric therapeutics, (4) anti-inflammatory therapeutics (i.e., microglia, astrocytes), (5) hormonal therapeutics, (6) synaptic therapeutics, (7) lifestyle therapeutics (e.g., regular exercise, yoga, meditation, diet), and (8) anti-sleep deprivation therapeutics.

## 2. Mitochondrial Therapeutics

The mitochondria are the major organelles responsible for a vast array of biochemical processes within eukaryotic cells, which have an outsized influence on the physiology of humans. Most famously mitochondria are known for ATP production via oxidative phosphorylation during cellular respiration [13]. However, mitochondria also play a crucial role in multiple essential metabolic pathways, Ca^2+^ homeostasis, apoptosis, and reactive oxygen species (ROS) consumption and production [14].

From a metabolic pathway perspective, pyruvate, which was oxidized from glucose during glycolysis, is converted to Acetyl-CoA by pyruvate dehydrogenase and enters the tricyclic acid cycle [15]. These pyruvates are subsequently combined with oxaloacetate, which creates citrate, a metabolic intermediate with more carbon atoms. These compounds are subsequently oxidized in the cycle, yielding 1 GTP, 3 NADH, 1 FADH2, and 2 CO_2_. NADH and FADH2 are both electron carriers essential for oxidative phosphorylation. NADH electron carriers generated from glycolysis can enter the mitochondria via the glycerol-3-phosphate or malate-aspartate shuttles [16]. Since the electrons carried by FADH2 are at a lower energy state than NADH electrons, they are transferred to complex II of the electron transport chain (ETC). Complex II of the ETC is also a succinate dehydrogenase of the TCA. Moreover, oxidative phosphorylation can be roughly viewed as having two major parts: the electron transport chain (ETC) and chemiosmosis [16]. The ETC is a collection of protein complexes bound to the inner mitochondrial membrane and organic molecules [17]. These electrons are passed through the ETC in a series of redox reactions, releasing energy. This energy is released in the form of a proton gradient, also called the proton motive force. It is this energy that is harnessed by chemiosmosis to make a large amount of ATP via the ATP synthase [16]. Most of this energy is dissipated as thermal energy or utilized to generate the proton motive force. The gradient increases the number of hydrogen ions within the intermembrane space (IMS), translating to higher acidity in the space and a charge differential between the IMS and matrix. The ETC proteins in general order are complex I, complex II, coenzyme Q, complex III, cytochrome C, and complex IV [16]. The complexes transfer their electrons to the following intermediates in the ECT: complex I to nicotinamide adenine dinucleotide (NADH)–coenzyme Q, complex II to succinate dehydrogenase–coenzyme Q, complex III to coenzyme Q–cytochrome c reductase, and complex IV to cytochrome c oxidase. The general flow of the ETC is summarized by the following list: (1) The electrons enter the ETC with NADH at complex I and then are transferred to complex II and coenzyme Q; from coenzyme Q, these electrons are then transferred to complex III, and then to cytochrome c. Next, the electrons passed to complex IV and finally to ½ O_2_, which makes H_2_O. As stated before, ATP is generated by using the electrochemical gradient created by the hydrogen pumping in these complexes to give H_2_O. Oxygen is a free radical; normal physiology dictates that only 1–5% of this oxygen is converted to ROS [18]. As a result, most of the intracellular ROS generation originates within the mitochondria. The production of mitochondrial superoxide radicals is thought to occur at primarily two primary points within the ETC, which are complex I (nicotinamide adenine dinucleotide dehydrogenase) and complex III (ubiquinone–cytochrome c reductase) [18,19]. Under most physiological settings, non-pathological ROS production is at complex III [18].

In pathological states, such as Alzheimer’s disease, high ROS production can damage NADH dehydrogenase, cytochrome c oxidase, and ATP synthase, causing a shutdown of mitochondrial energy production [18]. Regarding ion flow, normal mitochondria control Ca^2+^ fluxes across the membrane between intracellular compartments [18]. However, excessive ROS production can result in direct damage to Ca^2+^-regulating proteins [20]. Examples of these proteins are included in the following list: (1) the plasma membrane proteins (e.g., ligand- and voltage-gated Ca^2+^ channels), (2) endoplasmic reticulum Ca^2+^-ATP synthases [18], and (3) mitochondria electron-transport chain proteins. This ROS-induced disruption results in elevated Ca^2+^ concentrations. This increase in the relative level of free calcium ions ultimately disturbs Ca^2+^ homeostasis [18,19]. Additionally, the overproduction of ROS can damage the mitochondria. This mitochondrial damage often involves mtDNA mutations, mitochondrial respiration chain disruption, Ca^2+^ homeostasis alteration, and increased mitochondrial permeability [18,19]. Thus, it is logical to see why mitochondrial damage is suspected of playing an outsized role in the pathogenesis of neurodegenerative diseases [18].

Mitochondrial defects contribute significantly to neurodegeneration [21]. Studies indicate that mitochondrial dysfunction could be caused by the destruction of mitochondrial deoxyribonucleic acid (DNA) transcription [22]. Cells and tissues with high-energy demands, such as neurons (e.g., white matter in the brain), naturally contain more mitochondria [23]. AD and PD are associated with an increased risk of cardiomyopathy, which implies mitochondrial deficits might be more systemic [24]. One study investigated the disruption to the regulation of mtDNA-encoded gene expression in genes, such as platelet transmission electron microscope (PTEM)-induced kinase 1 (PINK1), PARKIN, and adenosine triphosphate (ATP) levels in peripheral mononuclear blood cells of PD [24]. This study indicated that the onset of disease for patients older than 50 was sporadic, but the onset of disease for patients under 50 only affected mitochondrial deoxyribonucleic acid (mtDNA) transcription [24]. However, only quality control genes were involved in familial PD cases, while the mtDNA copy number did not confound these data. Scientists also found evidence of elevated intracellular ATP levels in PD cases [25]. Gezen-Ak et al. assert there is systematic dysregulation in the control or regulation of specific mitochondrial DNA segments, which results in the sporadic formation of neurodegenerative disease. Researchers believe that ATP elevation might be the body compensating for the disorder [24].

**Figure 2:**
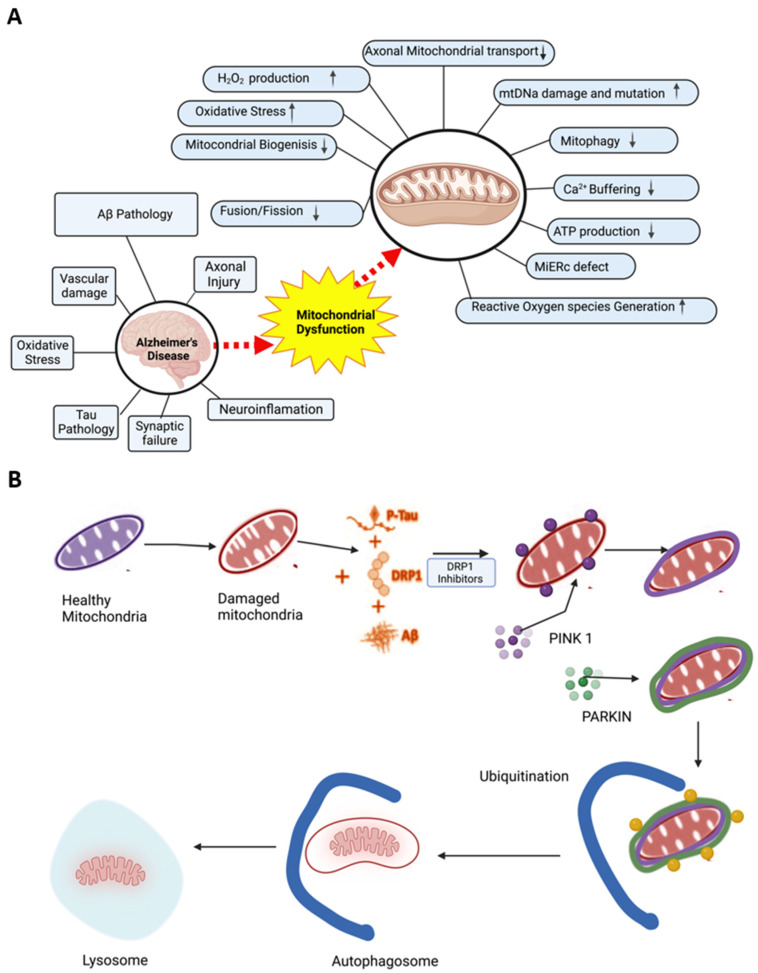
Mitochondrial dysfunction in Alzheimer’s disease and mitochondria-based therapeutics mechanism. (**A**) Mitochondrial dysfunction in the pathogenesis of AD results in the loss of mitochondrial structure and function that affects axonal transport, hydrogen peroxide production, mitochondrial biogenesis and dynamics, mitophagy, ER-mitochondria interaction, mitophagy, energy production, and ROS generation, as well as causing oxidative stress. (**B**) Mitophagy as a potential therapeutic target for treating dementia.

As a result, AD-related pathological changes (e.g., amyloid-beta deposition and neurofibrillary tangles) can occur. Because there is little-to-no neuronal regeneration in the brain, extensive damage to neuronal mitochondria is often devastating because mitochondria are primary sources/targets of oxidative stress [26]. Reactive oxygen species occur when electrons pass through the electron transport chain and leak out complexes I and III during oxidative phosphorylation. These electrons can react with oxygen and form superoxide anions via the enzymatic action of superoxide dismutase [27]. The presence of Fe^2+^ accelerates the decomposition of H_2_O_2_ to hydroxyl radicals and nitric oxide, which reacts with O_2_ to produce peroxynitrite. These compounds can play a role in mitochondrial dysfunction [27]. Refer to Figure 2 for an overview of the interplay between AD and mitochondrial dysfunction.

**Figure 3:**
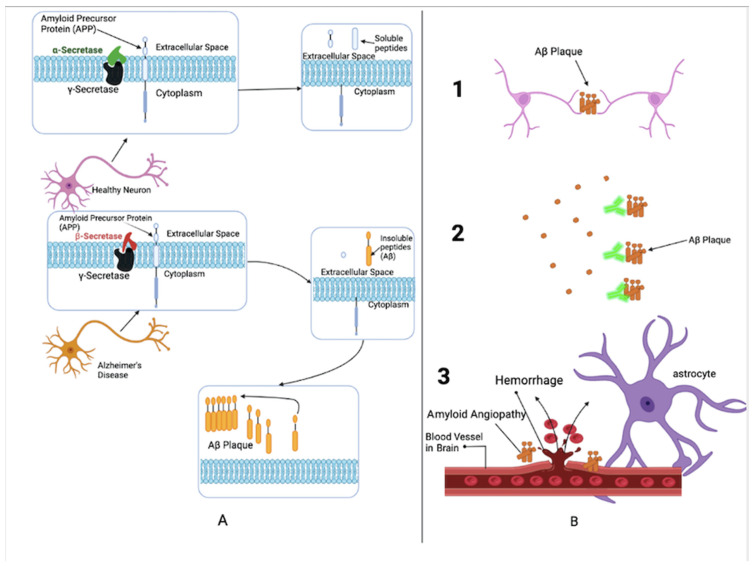
Amyloid-beta pathogenesis: (**A**) Normal (**Top**) and degenerate (**Bottom**) neurons, (**B**) Consequences of amyloid-beta plaque aggregation.

The mitochondrial defects mentioned in Figure 3 play a significant role in AD progression, but they may offer promising therapeutic targets for AD treatment and prevention. Researchers are currently studying how to restore mitochondrial function in AD patients via therapeutic antioxidants, including CoQ10 [28]; alpha-lipoic acid; and vitamins C, E, selenium, and glutathione [27]. In addition, several crucial enzymes, such as pyruvate dehydrogenase, alpha-ketoglutarate dehydrogenase, and cytochrome oxidase, are affected by antioxidants. Consequently, therapeutics that enhance mitochondrial bioenergetics reverse oxidative stress, or restore mitochondrial function may serve as novel AD therapeutics [29].

Ubiquinone (CoQ10), a common antioxidant, acts as an essential cofactor for the electron transport chain (ETC) within mitochondria. This biomolecule helps regulate oxidative stress, ROS formation, and inflammation [30]. Both in vivo and in vitro studies indicated that CoQ10 has a neuroprotective effect on cognitive functions. Therefore, many AD patients take ubiquinone to relieve symptoms. Alpha-lipoic acid, a coenzyme of mitochondrial pyruvate dehydrogenase and α-ketoglutarate dehydrogenase, is also an antioxidant responsible for recycling other antioxidants (e.g., vitamin C, E, selenium, glutathione) [27]. Researchers have also examined the part of dynamin-related protein 1 (Drp1) in malfunctioning mitochondrial fragmentation, autophagy/mitophagy, and neuronal damage in AD. One of the studies determined that Drp, part of a family of GTPase proteins, has been evolutionarily conserved to a high degree across many species. Drp1 is crucial for mitochondrial division, shape, size, and distribution throughout the neuron, from the cell body to axons, dendrites, and nerve terminals [6]. More specifically, differentially phosphorylated forms of Drp1 act to increase the fragmentation and/or increase the fusion of mitochondria. Moreover, elevated levels of Drp1 were found in diseased states, such as AD. Over the past twenty years, several Drp1 inhibitors have been developed, such as Mdivi-1. These inhibitors have also been tested on cell cultures and neurodegenerative mouse models. 

The Drp1 inhibitor Mdivi-1 has been found to inhibit excessive mitochondrial fission, promote mitochondrial fusion, and protect cells from mitochondrial dysfunction. Moreover, this inhibitor diminished the Aβ-induced synaptic depression in hippocampal cells. In contrast, the control group of transgenic mice showed a steep decline of approximately 50% in exocytotic synaptic vesicles. Additionally, Mdivi–1-treated mice showed inhibition of BACE1 gene transcription and translation [31]. In the same vein, the Reddy research group concluded similar results when investigating the effect of Mdivi-1 treatment on N2a cells with Aβ toxicity [32]. The research group found that the mouse neural crest-derived cell line, Neuro 2A (N2a), cells treated with Mdivi-1 had many effects. The treatment restored biogenesis, reduced peroxide production/oxidative stress, and lowered lipid peroxidation. The N2a cells’ mitochondria also demonstrated recovery cytochrome c oxidase activity and reduced fragmentation. The productive effects of Drp1 inhibition with Mdivi-1 were more significant for N2a cells pre-treated with Mdivi-1 than those treated with Mdivi-1 post-Aβ42 toxicity [6,32]. Mdivi-1 is considered for clinical trials for human diseases, such as AD, Huntington’s, and Parkinson’s.

Another study investigated the effect of combining Mdivi1 with Szeto–Schiller (SS) tetrapeptides, a group of tiny peptides, which act as antioxidants. These peptides can also cross the IMM and reach the mitochondrial matrix [33,34]. It was determined that SS31 diminished Aβ peptide production, mitochondrial dysfunction, and enhanced mitochondrial biogenesis and synaptic activity in AD mouse models [35]. The Reddy research group tested the combination of SS31 and Mdivi1 on cultured AD cells, generating positive results. These results suggest that a treatment regimen involving multiple different mitochondria-targeted antioxidants could have higher effectiveness in treating AD disease [36]. The scientific community must closely examine this treatment regimen as the results are promising. 

Coenzyme Q was considered one of the best possible therapies for AD in the past but never worked due to low bioavailability in the brain. As a result, mitoquinone mesylate (MitoQ) was created. It is a ubiquinone antioxidant compound conjugated with triphenylphosphonium (TPP). TPP functions to target the mitochondria because it accumulates on the negative side of the mitochondrial membrane, allowing it to effectively cross the lipid bilayers of the mitochondria [37,38]. This drug acts as a reactive oxygen species collector in AD model systems. It was determined that MitoQ reduced Aβ peptide levels, diminished ROS production/oxidative stress, and prevented synaptic loss and astrogliosis. MitoQ was also demonstrated to improve the cognition of transgenic mice [39,40]. MitoQ and other similar antioxidants, such as SkQ1, MitoApo, and astaxanthin, are worth investigating further by the scientific community for this drug’s potential use in treating AD [34]. 

Another possible mitochondrial therapeutic being considered for AD is oxaloacetate. Oxaloacetate is an intermediate of the Krebs cycle and gluconeogenesis. Studies involving AD mice revealed oxaloacetate treatment inhibited Aβ-mediated synaptic activity in the hippocampus [41]. More specifically, these studies indicated positive impacts on glycolysis, mtDNA, mtDNA-encoded proteins, respiratory fluxes, activation of mitochondrial biogenesis, neuroinflammation, and insulin signaling changes in brain activity [42]. However, while the preclinical results were promising, clinical trial results posted in 2021 indicated that the treatment did not significantly impact/improve cognition in a meaningful capacity [34]. A list of current ongoing trial therapies based on mitochondrial complex I inhibitors and others are listed in Table 1.

## 3. Anti-Amyloid-Beta Therapeutics

Alpha- and γ-secretase enzymes usually digest amyloid precursor protein (APP) to produce soluble proteins that can be broken down and recycled later by the cell [43,44]. In the pathological setting of AD, the gamma-secretase works in tandem with the beta-secretase to create small peptides, forming protein aggregate plaques [45] (see Figure 3). These small peptides are scientifically known as amyloid-p (Aβ) peptides. The peptides are cleaved by β- and γ-secretase enzymes from the integral membrane protein, amyloid precursor protein [46]. These Aβ peptides usually accumulate in the brain, form Aβ plaques (ABP), and act prion-likely [47]. Studies suggest that Aβ peptides regulate synaptic plasticity, and the production of such Aβ peptides causes the pathology related to ABPs. The formation of ABPs in the brain causes the development of multiple issues. Extensive research identifies three primary effects: (1) neuronal signaling disruption, (2) inflammation of the neurons, and (3) angiopathy [47].

When ABPs lodge themselves in the synapses of neurons, neurotransmitters have limited travel between the synaptic cleft and healthy neurons. When these neurons fail to signal, neurons and the brain become damaged, which alters brain functions, such as memory and cognition. ABPs can trigger immune responses in the body leading to localized inflammation that can damage nearby neurons. The deposition of ABPs in the brain on extracellular surfaces of blood vessels causes angiopathy and can ultimately rupture and cause bleeding of blood vessels [47].

### β-Site Amyloid Precursor Protein Cleaving Enzyme 1 (BACE1) Inhibitors

A significant area of research surrounding AD therapeutics involves BACE1 inhibitors. This therapeutics target the beta-secretase enzyme responsible for the cleaving amyloid precursor protein or the beta-site APP cleaving enzyme 1 (BACE1) [48]. Because BACE1 is the principal secretase, scientists created transgenic mouse models with overexpression of human APP with known familial AD mutations and a germline gene deletion of BACE1 to test the effect of deletion on ABP generation in mice prone to developing AD. These studies found that knocking out the beta-secretase enzyme prevented any meaningful amyloid-beta plaque generation/accumulation, which indicates that BACE1 inhibition has a direct effect on ABP formation [48,49]. Furthermore, the inhibition of BACE1 also stated the following: (1) a rare human mutation to BACE1 reduced ABP formation by approximately 40% and reduced the rate of AD development 5- to 7-fold [49], (2) the limitation of the number of BACE1-containing vesicles in the active zones of synapses directly reduced the effect of ABPs on synaptic transmission and communication [49], (3) gene deletion of BACE1 in mouse models prevents memory deficit and neuronal loss [50], and (4) the inhibition of gamma-secretase is not feasible due to its essential physiological functions beyond its role in the pathological formation in ABPs [47,51].

These findings warrant continued BACE1 inhibitor research, development, and trials to determine the potential for BACE1 inhibitors to inhibit ABP formation in the brain. BACE1 likely has a multifactorial impact on normal physiology beyond what is currently understood in the literature. Research studies conclude that BACE1 has a well-known effect on myelination by cleaving and activating Neuregulin-1 [48]. This function of BACE1 makes this enzyme critical for early postnatal development of the peripheral nervous system (PNS); however, further research needs to be done to elucidate specific details on the effect of BACE1 on the PNS. Like many rate-limiting enzymes, BACE1 is regulated by a variety of post-translational mechanisms, including the following sites: (1) 5’UTR, (2) microRNA, and (3) non-coding sense RNA [48,51]. Some BACE1 inhibitors have been developed to allosterically module BACE1 via monoclonal antibodies [52]. BACE1 inhibitors can cross the blood–brain barrier (BBB) to the brain due to their small size [49]. It is challenging for researchers to make BACE1 inhibitors highly potent and specific due to their small size.

In the field of research on Alzheimer’s disease, a conflict has existed for many years. The amyloid hypothesis supporters argue that an aberrant buildup and aggregation of amyloid-beta is a major factor in setting off a series of pathological occurrences that result in the clinical symptoms of AD. On the other hand, opponents argue that amyloid deposition is an epiphenomenon that has diverted attention away from the real causes of AD, which are still mostly unknown [53]. Many studies also showed metastable oligomers composed of small numbers of Aβ monomers are the neurotoxic culprit, with amyloid plaques perhaps acting as a reservoir for such species. Now that amyloid-beta plaque-removing drugs for AD are available, the dispute over the amyloid hypothesis has gone from the lab to the clinic, as shown in Table 2. In clinics around the country, the actual clinical trial is currently underway [53].

**Figure 4:**
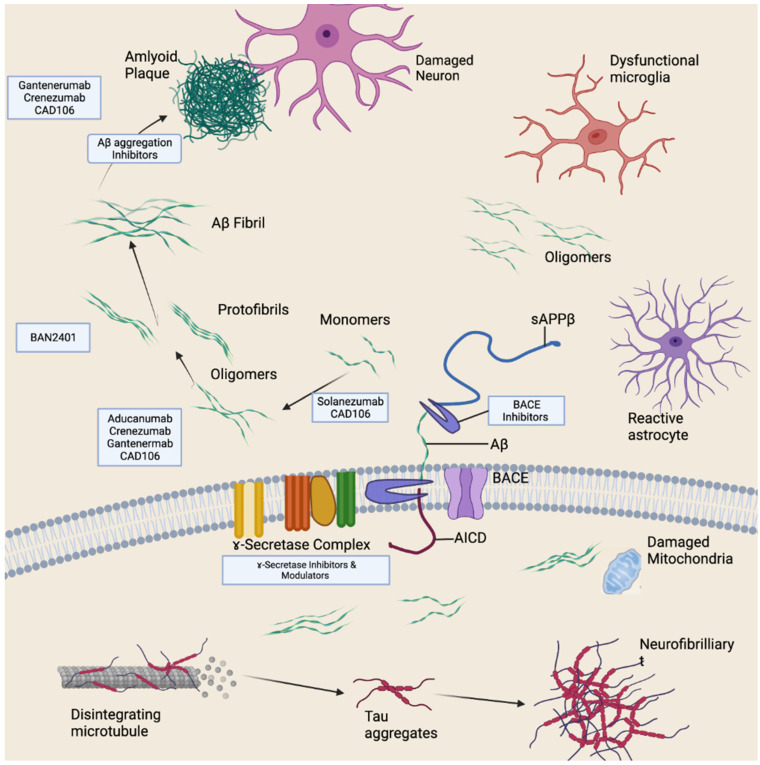
Anti-amyloid-beta therapeutics. Therapeutic strategies target different forms of amyloid beta to varying stages of its processing.

Many amyloid-beta-targeting drugs have proved to be clinically ineffective. The FDA has granted accelerated clearance to aducanumab, one of four anti-amyloid-beta antibodies that have been demonstrated to mediate the removal of amyloid plaque from the brains of AD patients [54]. By specifically attacking amyloid-beta oligomers and fibrils, the monoclonal antibody aducanumab, which was recently licensed by the Food and Drug Administration (FDA), was able to diminish Aβ accumulation and prevent the course of cognitive impairment [55]. Many other drugs are in development and trials, as shown in Figure 4. However, experimental drug trials (see Table 2) warrant further discussion.

## 4. Monoclonal Antibodies

Scientists are currently researching monoclonal antibodies, which are artificially produced antibodies designed for specific target molecules (e.g., lipids and proteins) [56]. Single-domain antibodies (VHHs) are potentially disruptive therapeutics [57]. They have many applications in the medical field, especially with neurological disorders. However, scientists have not used VHHs much in research on the central nervous system (CNS) due to their difficulty crossing the BBB [58]. A recent study describes a gene transfer strategy based on BBB-crossing adeno-associated virus (AAV)-based vectors designed to deliver VHHs directly into the CNS of mammals. Researchers used this vector to provide VHHs to inhibit BACE1 in the brain for exploratory therapeutic research. Results showed the VHH developed, called VHH-B9, to be highly selective for BACE1. Furthermore, researchers concluded that a single systemic dose of VHH-B9 produced long-term benefits (e.g., improvement in cognition) in mice for up to 12 months [58]. Regarding efficacy, research results from experiments on amyloid precursor protein (APP) mouse models are fascinating and promising.

According to another study, prime apes infused with anti-BACE1 antibodies via an intracerebral (i.c.v.) showed a strong, clear, and sustained reduction of up to 70% in the cerebrospinal fluid’s (CSF) amyloid-β (Aβ) peptides [59]. Despite initial concerns about antibodies crossing the BBB, antibody distribution (ranging from 20 to 40 nM) was nearly uniform in brain parenchyma because antibodies were directly injected into the CNS. Results showed approximately a 50% reduction in Aβ in the cortical parenchyma. In contrast, nonhuman primates that received anti-BACE1 intravenously showed no statistically significant change in cortical or CSF Aβ levels. These animals also had a low (~0.6 nM) antibody concentration in the brain [59]. Unfortunately, the VHH-B clinical trial was determined to be unsafe for humans. The trial was terminated in 2021 due to unexpected changes in cognitive function, brain volume loss, and body weight loss, according to clinicaltrials.gov.

### Anti-Tau Therapeutics

**Figure 5:**
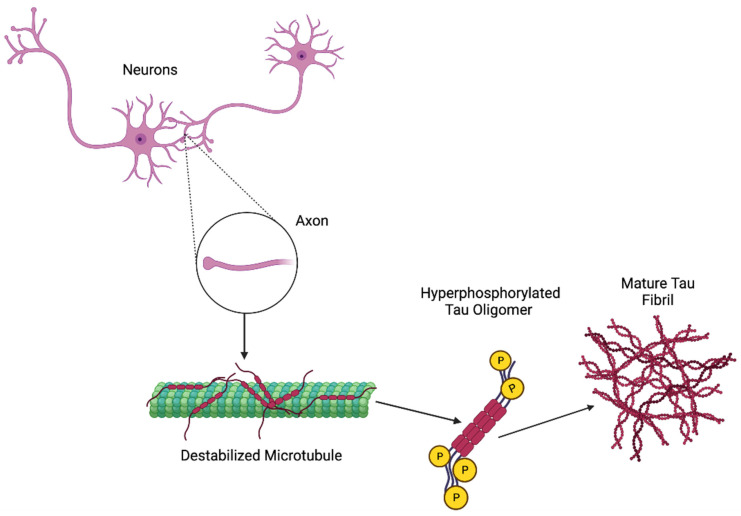
Possible pathogenesis mechanism of tau.

Tau is a microtubule-associated protein that helps the brain stabilize axonal microtubules, but an abnormal deposition of this protein leads to various diseases, including dementia [60]. The pathogenesis of tau is represented in Figure 5. Current therapeutics, as presented in Table 3, for tau treatment include targeting multiple forms of intracellular and extracellular tau to prevent pathological tau formation, accumulation, and spread [61]. However, various strategies include the inhibition of protein kinase, the inhibition of tau aggregation, antisense oligonucleotides (ASOs), and active and passive immunotherapies [61]. Most small molecules that target tau are aimed to directly or indirectly modify its aggregation [62]. Table 3 represents the list of currently ongoing clinical trial therapeutics of tau.

## 5. Tau Immunotherapies

The effectiveness of tau immunotherapies was first reported in the JNPL3 mouse model in 2007, which showed remarkably less tau pathology in different brain regions [63]. Currently, most tau-targeted therapies in preclinical trials are immunotherapies [60].

### 5.1. Passive Immunization

Passive immunization includes the administration of monoclonal antibodies [60].

JNJ-63733657: This humanized IgG1 monoclonal antibody recognizes the temperate region of tau [64]. Accumulating evidence suggests these monoclonal antibodies can potentially prevent tau’s cell-to-cell propagation and aggregation [65]. Janssen Research and Development ran two clinical trials in phase 1 to evaluate its safety and tolerability in participants [66]. The 2017 phase I clinical trial reported the monoclonal antibody to be safe and well tolerated [66]. In 2019, a phase I clinical trial reported dose-dependent increases in exposures. CSF exposures were ∼0.2% of serum levels and dose-dependent reductions in p217+ tau in CSF [67]. In January 2021, a phase 2 study was commenced into 420 patients with early AD symptoms and a positive tau PET scan, and the trial will last until 2025 [68].

UCB0107: This humanized, monoclonal IgG4 antibody has an affinity to binding to paired helical filaments of tau and it recognizes amino acids 235–250 near tau’s microtubule-binding domain [65,69]. In March 2019, the company completed a phase 1 trial in 24 healthy Japanese men who received a single, unspecified UCB0107 dosage or placebo [64]. The endpoints were adverse events and pharmacokinetics. The phase 2 trial plans to randomize 450 people with mild cognitive impairment or mild AD dementia to one of two doses of UCB0107 or a placebo for 80 weeks. This trial will last until 2025 [68].

### 5.2. Active tau-Targeted Vaccines

Presently, two anti-tau active vaccines are in a clinical trial.

ACI 35: This liposome-based vaccine contains 16 copies of synthetic tau fragments phosphorylated at phosphorylation sites at S396 and S404 and is anchored into a lipid bilayer to activate the immune system to produce antibodies [60]. In July 2019, AC Immune and Janssen started a small phase 1b/2a trial to test the safety and immunogenicity of ACI-35 in people with early AD. In July 2020, AC Immune announced that the lowest-dose cohort was completed, and ACI-35 had generated favorable safety, tolerability, and immunogenicity data. In February 2022, ACI 35 phase 1b/2a trial interim data confirmed consistent the safety and potent immunogenicity of the pTau Alzheimer’s vaccine in a high-dose cohort in early AD.

AADvac-1: This is an active vaccine designed to elicit an immune response against a pathologically altered form of tau pathology [70]. This first-generation active immunotherapy vaccine targets 12 amino acid sequences in the tau protein’s microtubule-binding region (MTBR) [71]. In May 2013, Axon neuroscience started a phase 1 trial in 30 patients with mild-to-moderate AD. This vaccine was highly immunogenic in humans, inducing IgG antibodies against the tau peptides in 29 patients out of 30 [70]. In this study, overall, AADvac -1 was safe and tolerable.

The phase 2 trial was conducted in 196 patients with mild-to-moderate AD, as per NIA-AA. Eleven doses of AADvac were administered to patients at 40 μg per dose throughout the trial. Participants were randomized 3:2 to receive a dose and placebo. The problem met its primary objective of safety and tolerability, while the secondary objectives were to evaluate the immunogenicity and efficacy of treatment [72]. Criteria and therapy met its primary endpoint.

### 5.3. Tau Aggregation Inhibitor

The first direct tau aggregation inhibitor is methylene blue, also known as methylthionine chloride [64]. Derivatives of methylene blue have revealed a specific potential to inhibit tau aggregation [73]. TRx0237 (LMTM) is the second-generation tau aggregation inhibitor (TAI) for AD and FTD and is the purified form of methylene blue. Wilcock GK and colleague’s study on examining the efficacy of LMTM as monotherapy in non-randomized cohort analyses as modified primary outcomes in an 18-month Phase III trial in mild AD support the hypothesis that LMTM could be effective as monotherapy and that the administration of a 4 mg dose twice a day may serve as well as higher doses [74]. In a phase 3 trial, LMTM could not slow cognitive or functional decline in mild-to-moderate AD. Still, after re-investigating the data, the brain atrophy rate of patients with LMTM as monotherapy was remarkably lower than that of mild AD [68]. A phase 3 trial of a lower dose is in progress and will be complete in December 2022 [68].

## 6. Anti-Inflammatory Therapeutics

The significant risk factors that affect AD pathogenesis include hyperphosphorylation of tau protein, Aβ accumulation, and neuroinflammation. Neuroinflammation’s considerable role in AD progression can be controlled by anti-inflammatory drugs [75]. Various proteomics studies have shown that neuroinflammation is caused by an abundance of interconnected irregular metabolic pathways triggered and propagated by Interleukin-1β (IL-1β), tumor necrosis factor-alpha (TNF-α), transforming growth factor beta (TGF-β), and triggering receptor expressed on myeloid cells 2 (TREM2) [76]. Moreover, microglia and astrocytes, important for neuroinflammation under physiological conditions, are critical drivers of neuroinflammation [76]. Therefore, the potential therapeutic target for alleviating neuroinflammation may include TNF-α, TREM2, and myeloid cell surface antigen CD33 [77]. Dominant-negative TNF inhibitors, such as XPro^®^1595 [67] and XENP 345 [68], are considered effective therapeutics for targeting TNF.

AL002, a humanized monoclonal IgG1 antibody, is an anti-human TREM2 antibody under phase 2 study; phase 1 study data suggested it to be safe and tolerable by healthy adults [78]. The neuroinflammatory treatments with anti-CD33 antibodies are also promising as CD33 is involved in the inflammatory response. The anti-CD33 antibodies lintuzumab and gemtuzumab ozogamicin are effective therapies [79] as lintuzumab has been reported to the microglial cell surface CD33 by 80% [80]. CD 33, a transmembrane receptor with anti-inflammatory signaling function [81], is predominantly found on the surface of immune cells, such as myeloid progenitor cells, macrophages [82], monocytes, and dendritic cells [83], while in the brain CD 33 is precisely expressed by infiltrating macrophages and microglia. CD 33 controls inflammatory responses of the innate immune system [82]. Infiltrating monocytes play a constructive role, probably by eliminating amyloid plaques [84]. The expression of CD33 is increased in the AD brain, where it is thought to control the activation of microglia and reduce microglial uptake and clearance of Aβ [85]. The CD 33 antibodies reduce CD 33 protein levels mostly by stimulating internalization and breakdown or by decreasing CD 33 action [86]. The ability of microglia to clear Aβ appears to be regulated by the surface expression of CD33 and alternative splicing, possibly due to D2 domain-dependent activation [82,87,88,89]. These interventions as immunotherapy may represent the main strategies for treating dementia.

## 7. Hormonal Therapeutics

### 7.1. Purpose of MHT for Dementia

The realm of research on hormonal therapeutics for menopausal women is noteworthy. Women often take menopausal hormone therapy (MHT) drugs, also called hormone replacement therapy (HRT) drugs, to replace certain hormones (i.e., estrogen, progesterone) produced by their ovaries that are lost during the menopausal transition. MHT helps alleviate symptoms of menopause and future problems associated with hormone decline during and after menopause [90]. MHT therapeutics are comprised of numerous types of estrogen, such as endogenously produced hormones, including estradiol, estriol, and other estrogenic compounds, such as the commonly prescribed conjugated equine estrogen (CEE). Although these hormones are not identical, their effects on the human body are similar [91]. It is a well-established fact that women account for most AD [92]. Scientists believe women may develop AD at higher rates than men because: (1) women live longer, (2) women develop more autoimmune conditions, and/or (3) women lose sex hormones during menopause [7]. Most research on MHT focuses on longitudinal, multiyear studies on the effects of MHT on menopausal women. 

### 7.2. Early Research on MHT and Dementia

Early research studies produced inconsistent results regarding the association between MHT and dementia. In 2005, the Zonderman group conducted longitudinal research that indicated women receiving MHT had a reduced risk of incidence of AD, as compared to the control group [93]. However, this 2005 study found MHT did not significantly prevent the development of dementia or have a therapeutic effect on the cognition of elderly menopausal women already experiencing AD symptoms [93]. In stark contrast, a randomized controlled trial persuasively showed that estrogen and progestin therapy doubled the risk of progressing dementia, in comparison to the placebo group among women aged 65 or older. This specific study also indicated the lack of benefit to those with mild cognitive impairments [94]. According to the Women’s Health Initiative Memory Study, estrogen plus progestin therapy had a positive effect on figural memory but a negative impact on verbal memory [95]. A subsequent study by the same group of researchers indicated that estrogen-alone therapy demonstrated no significant preventative effect on the development of dementia [96]. In addition, twenty consecutive weeks of estradiol-alone MHT did not improve cognition in women aged 70 or older [97].

### 7.3. Recent Research on MHT and Dementia

Various studies implicate the neuroprotective effects of estrogen [98]. On a cellular level, estradiol is a sex steroid and a neuro-steroid locally present or synthesized in the brain. Scientists believe both hormonal and brain-synthesized estrogens are neuroprotective because brain-synthesized estradiol regulates adult neurogenesis, synaptic plasticity, cognition, and other biological effects [99]. In pathological states, the enzyme of aromatase (which produces estradiol) is upregulated and induced in astrocytes, serving as a neuroprotective mechanism [100]. The silencing of brain aromatase is linked to increased neurodegeneration. As with most steroid hormones, estradiol enters the cell and binds to a receptor within the cell’s nucleus to modulate gene expression/transcription via estrogen receptors, ERα and ERβ.

Additionally, estradiol has receptors on the cell surface, including the following list: Gαq protein-coupled ER, G-protein-coupled ER, ERα, and ERβ. These receptors coordinate neuroprotective mechanisms, but this signaling can also be redundant [100]. Other nuclear regulatory receptors regulate the intranuclear transcription factors via diminished transcription. In addition, various protein kinases regulate surface estrogen receptors, which inactivate the receptors via phosphorylation. These extracellular and intracellular receptors also highlight the interaction of estradiol with other neuroprotective substances, such as wingless and Int-1 (WNT), Notch, insulin-like growth-factor-1, and brain-derived neurotrophic factor [100]. 

A recent meta-analysis described the results from multiple original articles on HRT and dementia development. Researchers concluded that menopausal hormonal therapy (MHT) might reduce the risk of AD by approximately 11–33% [101]. However, the risk reduction may substantially vary from the time MHT is initiated. This study indicated that MHT benefited some risk factors, augmented the function of some protective factors, and failed to improve the cognition of women with dementia [101]. See Table 4 for a summary of this meta-analysis. 

Yu-jin Kim and colleagues’ study on menopausal hormone therapy impact and risk of neurodegeneration disease, including AD, found hormonal therapy was linked with remarkably reduced risk for combined neurodegenerative disease [102]. They also found a protective effect with a greater duration of therapy, compared to short-term therapy [102].

J. E. Yoo and colleagues’ study on female reproductive factors linked with dementia, found the use of hormone replacement therapy and oral contraceptive independently linked with the incidence of all-cause dementia AD and VaD. This study also demonstrates that all hormonal replacement therapy users had approximately 15% reduced risk of dementia, while all oral contraceptive users had approximately 10% lower risk of dementia [103].

Minjung Han and colleagues’ study on the association of tibolone and dementia risk in Korean women aged 50–80 years found the use of TIB (menopausal hormone therapy medication) does not have a remarkable association with total dementia risk [104].

Paganini-Hill and colleagues’ study to investigate the association of endogenous and exogenous estrogen exposure with the risk of incident dementia in a sample group over the age of 90 found prior exposure to estrogen either exogenously or endogenously had little effect on dementia risk in the 10th decade of life. They also observed that women with a high endogenous estrogen exposure index had a non-significant 25% lower risk [105].

## 8. Synaptic Therapeutics

The accumulation of Aβ monomers, oligomers, and deposits/plaques interfere with neuron-to-neuron communications at synapses that contribute to neurodegeneration [106]. When considering the inside of a neuron, tau tangles block the transport of neurotransmitters between the pre-and post-synaptic neurons [107]. Studies indicate that p-tau and Aβ oligomers have profound consequences on synaptic function, often resulting in subsequent dysfunction. Although there is limited research on the death of neurons, studies indicate that tau is associated with increased Aβ in the synapses [108]. Tau and Aβ plaques are believed to be toxic to neurons by activating microglia. These are the primary immune cells in the brain and are responsible for clearing harmful toxins (such as tau and Aβ) and dead cells [109]. In a pathological state, microglia cannot perform their cleaning duties, which causes chronic inflammation in the brain. This chronic inflammation is a valuable biomarker for AD, meaning its presence can be used to indicate AD’s presence or lack thereof [110].

Researchers are investigating the relationship among microRNAs (miRNAs), synaptic dysfunctions, mitochondrial dysfunctions, and AD development. These factors contribute to memory loss, impaired neurotransmission, and many other clinical symptoms associated with AD. Kumar and Reddy identified a clear association between the deregulation of miRNAs with synaptic and mitochondrial dysfunctions in AD [111]. Therefore, further investigation should be conducted on miRNAs as biomarkers or treatments for AD because of their potential involvement in AD.

Very few publicly disclosed drug trials with endpoints explicitly focus on synapse density/function [112]; however, AZD0530 and CT1812 (Elayta) are two therapeutics of interest listed in Table 5. These experimental trials are part of the following list: (1) LUCIDITY (NCT02546001), (2) NCT02167256 clinical trial, (3) NCT03493282 clinical trial, and (4) a clinical trial that targets the BDNF signaling pathway using natural products.

### 8.1. LUCIDITY Trial NCT02546001

The purpose of the LUCIDITY clinical trial (NCT02546001) is to study the effect that a methylene blue derivative has on synaptic function using fluorodeoxyglucose-positron emission tomography (FDG-PET) and cognitive endpoints. No official results have been posted on the outcome of this specific methylene blue derivative and TRx0237 [113]. Historically, methylene blue (methylthioninium chloride) has been used as a Food and Drug Administration (FDA)-approved treatment for methemoglobinemia, prevention of urinary tract infection (UTI) in the elderly, and a dye for intraoperative tissue visualization. Methylene blue is a tricyclic phenothiazine drug and dye, which acts as an inhibitor of guanylate cyclase and nitric oxide synthase.

Early results in animal models during phase II clinical trials demonstrated that methylene blue and TRx0237 might have a neuroprotective effect on the synapses with mixed efficacy [114]. Some results indicated only better cognitive outcomes if treatment was administered before the onset of AD symptoms and little effect on animals already presenting with AD symptoms. However, some research indicated that methylene blue and TRx0237 could reverse cognitive deficits [115]. The mixed results from animal models with methylene blue and TRx0237 suggest the treatment may: (1) increase proteasome activity, (2) increase autophagy, (3) reduce tau levels, (4) reduce amyloid beta plaques, and (5) improve cognition. More specifically, methylene blue is thought to reduce oxidative stress in androgen-binding proteins (ABP) and phosphorylated tau in AD when tested in an in vitro model [116]. In vitro, methylene blue reduces free radical production and limits mitochondrial superoxide formation [117]. As a result of these reactions in the mitochondria, methylene blue has many downstream effects on metabolism, such as lipid β-oxidation, glycolysis, adenosine triphosphate (ATP) synthesis, extracellular matrix (ECM) production, and Na+/K+ ATPase activity. These metabolic effects increase the oxidative capacity in neurons. In addition, methylene blue’s structure can quickly oxidize the ETC in the mitochondrial matrix, making the compound an electron transporter. Methylene blue consumption also increases cellular oxygen use, energy production of ATP, and glucose uptake in primary astrocytes [118].

According to the Alzheimer’s Drug Discovery Foundation, trial results indicate low methylene blue doses, and TRx0237 shows some cognitive benefits. In a phase 3 study, 891 patients with mild-to-moderate AD patients have prescribed 75 mg or 125 mg of TRx0237 twice per day or the placebo, consisting of 4 mg of TRx0237, to control urine discoloration for 15 months. Unfortunately, the drug was ineffective in its impact on cognition or function. However, a secondary analysis of patients taking the drug as a monotherapy revealed significant benefits in cognition (ADCS-CGIC, ADAS-Cog, MMSE) and function (ADCS-ADL) and reduced lateral ventricular volume (Gauthier et al., 2016). As a result of these findings, this reduced form of methylene blue is now a potential monotherapy for future clinical investigations. In addition, researchers subsequently statistically modified the same phase 3 clinical trial to compare the effects of 100 mg TRX0237 twice per day with a placebo (4 mg TRX0237 twice/day), as well as the placebo (4 mg TRX0237) as a monotherapy to a TRX0237 as an add-on therapy [119].

Researchers conclude that patients taking the TauRX0237 monotherapy experienced less decline in cognition, function, and glucose uptake than patients who took the TRx0237 as an add-on therapy. Moreover, AD patients taking 100 mg showed more benefit than those taking 4 mg; however, it should be noted that the 4 mg group had both monotherapy and add-on patients combined in the analysis. Nevertheless, these results suggest that TRX0237 should be investigated as monotherapy for AD.

### 8.2. Clinical Trial NCT02167256

The main focus of clinical trial NCT02167256 is to monitor the effects of AZD0530, a kinase inhibitor Src/Abl tyrosine kinase inhibitor saracatinib, on synaptic function with FDG-PET and cognitive endpoints repurposed for the treatment of AD in this clinical context. The research team investigated if oligomeric amyloid-β peptide binding to cellular prion protein on the neuronal cell surface activated intracellular Fyn kinase. This kinase would subsequently mediate synaptotoxicity and tauopathy [120].

In terms of the study participants, all 159 who had mild AD and elevated ABP levels in the brain were given AZD0530 (100 mg or 125 mg daily) vs. placebo for 52 weeks. Participants experienced adverse side effects (e.g., gastrointestinal disorders with diarrhea being most prevalent), which caused the cessation of treatment. These negative effects occurred in 38 participants (48.1%) who received AZD0530 and 23 (28.8%) with the placebo. Statistically significant results of AZD0530 treatment were not found on relative cerebral metabolic rate for glucose (CMRgl) reduction in an AD-associated region of interest or on secondary clinical or biomarker measures [120].

### 8.3. Clinical Trial NCT03493282 (SPARC)

The SPARC clinical trial, NCT03493282, is designed to assess the effect of Elayta and an SV2a PET ligand UCB-J on sigma2 receptor antagonists CT1812 on patients with mild-to-moderate AD [112]. Elayta, designed to be a small-molecule antagonist of the sigma2 receptor, binds to the progesterone receptor membrane component 1 subunit of the receptor complex [121]. In the SPARC trial (NCT03493282), scientists administered 100 mg CT18127 to seven randomized subjects, as well as 300 mg CT1812, and seven subjects were randomized to a matching placebo as treatment. There were reportedly no statistically significant differences between the placebo and experimental groups regarding synaptic density. Researchers believe Elayta could be used as a therapeutic to the affinity for oligomeric Aβ for its receptor because it interferes with Aβ-induced synaptic toxicity.

The drug CT1812 grew out of screening programs conducted at Cognition Therapeutics. Company scientists claim compounds in this series block the binding of a host of different Aβ species to neuronal receptors and displace Aβ species from neuronal receptors [122]. Although scientists at Cognition Therapeutics have not disclosed the structure of Elayta, researchers report that similar compounds can cross the blood–brain barrier (BBB), occupy up to approximately 80% of sigma2/PGRMC1 receptors, and restore behavioral deficiencies in amyloid precursor protein (APP) transgenic mice [122]. In preclinical work, Izzo et al. found that CT1812 helped: (1) clear neural Aβ oligomers, (2) improve cognition in APP mice, and (3) displace Aβ oligomers from human postmortem AD brain tissue [123]. According to Cognition Therapeutics scientists at the Alzheimer’s Association International Conference on 4 August 2022, Elayta has been well tolerated in clinical studies; however, mild, and transient elevations of liver enzymes have occurred among patients. Refer to Table 5 for synaptic therapeutics for dementia.

## 9. Lifestyle Therapeutics

Several lifestyle therapeutics are available to treat patients with dementia, including physical exercise, yoga, meditation, and diet. Studies conducted during the past 10 years indicate that physical activity helps protect against the development of dementia. Observational studies found that older physically active individuals experience less cognitive decline and the subsequent development of dementia [124]. Although physically active adults have less risk of developing AD, studies are not clear about the effect of exercise on the development of vascular dementia. In addition, there is a need for research on the impact of exercise on middle-aged adults and their risk of developing dementia [125]. However, the study correlates higher levels of physical activity in older adults with a reduced risk of developing all forms of dementia, including AD [124].

## 10. Physical Exercise

Interventional studies find that exercise training can improve cognitive performance even for a short duration. Exercise may also reduce the level of cognitive decline in people with cognitive impairment [124,126,127]. In addition, meta-analysis studies found that sedentary individuals experience improvements in their cognitive abilities in as short a period as four months. These findings indicate that doctors, hospitals, and care facilities may utilize physical exercise in their treatment of dementia patients soon.

The effect of physical activity on a cellular level is complex and comprised of several factors. According to several studies, adults who are more physically active have higher levels of neurotrophic factors in the brain, which are associated with neurological repair [128,129]. Adults with better cardiovascular health from physical activity have a decreased risk of developing vascular dementia and AD. Animal models also indicate that rats with high levels of physical activity have less β-amyloid plaque formation in their brains upon examination [126,128]

### 10.1. Yoga

Yoga is considered a mind-and-body practice for good mental and spiritual health. Although there is little empirical data on the therapeutic effects of yoga on dementia patients, eight studies found that dementia patients with mild cognitive impairments benefit from yoga as a primary intervention or as part of a multi-system approach to treatment. These studies indicate beneficial effects on verbal memory, most significantly on attention, sleep, mood, neuronal connectivity, and general cognitive functioning. Although studies on yoga as a therapeutic are limited, healthcare providers may consider recommending yoga to patients with AD with mild cognitive impairments as a safe and potentially helpful additional treatment [130].

### 10.2. Meditation

Mediation has recently been looked at as a treatment for those who have dementia. Meditation is the act of one engaging in contemplation and reflection before making decisions. Many also see it as a mental exercise with an innately spiritual purpose. The act of meditation often gives individuals a sense of calm, peace, and balance in their lives [131]. For research, secular mindfulness-based meditation programs are often the focus.

According to a recently published meta-analysis, meditation-based interventions were found to positively impact the quality of life, mental health, cognition, and functional abilities of patients with AD [132]. Although these results are exciting, it is essential to consider the flaws in these conclusions when considering weak research design, inconsistency in outcome measurements, small sample sizes, and overall feasibility/lack of standardization of mediation practices for individuals with dementia [132].

### 10.3. Diet

Studies indicate that nutrition plays a role in the development of AD [133]. Although dietary recommendations are not a treatment for AD, a healthy diet may modulate the symptoms experienced by AD patients and slow their cognitive and physical decline [134]. The Mediterranean diet (MD) includes multiple nutrients that correlate with a decreased risk of developing AD or mild cognitive impairment [135,136]. The nutrients emphasized in the MD have the antioxidant properties of olive oil, wine, vegetables, fruits, vitamins, and polyphenols. These substances are theorized to have a neuroprotective effect due to their ability to reduce oxidative stress and inflammation [136,137]. Literature reviews indicate that the MD directly or indirectly influences the factors that modulate the neurodegeneration seen in AD [133].

### 10.4. Anti-Sleep Deprivation Therapeutics

Neurodegenerative diseases are commonly linked to sleep disorders [138]. Many studies have shown that sleep disturbances may lead to cognitive impairments due to AD [139]. Although good sleep plays a significant role in restoring brain functions and retaining memory, sleep is a critical factor in AD progression. The evidence of various studies has shown that sleep leads to changes in cell structure and affects the Aβ clearance mechanism [140]. Alzheimer’s patients show a significant disturbance in their circadian sleep–wake cycles, compared to similar-aged healthy individuals. The breakdown of the circadian sleep–wake cycle gets worse with the progression of the disease. The sleep architecture in AD patients is characterized by a further decline of rapid eye movement (REM) sleep, slow wave sleep (SWS), and an increase in the frequency and timing of awakening patterns, as compared to same-aged control individuals [141]. Donepezil has shown increased REM sleep and decreased REM latency in Alzheimer’s patients [142].

Melatonin, a hormone produced in the pineal gland, regulates the circadian sleep–wake cycle and has neuroprotective effects against tauopathy [143]. A case study reported in 2019 showed improved sleep time and preventive effect on delirium in elderly AD patients by treating them with suvorexant, an orexin receptor antagonist. Suvorexant enhances cerebral orexin activity that might be linked with sleep disorders due to delirium in elderly AD patients [144].

## 11. Conclusions and Future Directions

Dementia itself is not a disease. Instead, this general term refers to the cognitive decline and psychological changes caused by several neurodegenerative conditions. Dementia has many social, economic, psychological, and physical impacts on patients and their caregivers. The prevalence of dementia is increasing rapidly in the elderly population. More specifically, females are more likely to develop dementia than males due to oxidative stress in females experienced during menopause, effectively creating more amyloid-beta plaques in the brain. Studies have shown that females are at higher risk of developing AD, as compared to men who are comparatively at greater risk of developing vascular dementia. Gender is not the only discriminating factor in the development of dementia pathology; ethnicity also affects the prevalence of the disease. The rate of dementia and AD is higher in African Americans and Hispanics, as compared to the White population in the United States. The consideration of such discriminatory factors in the development of dementia pathology are important biological variables in research focusing on dementia and can help in advancing the overall understanding of the pathology and treatment of dementia. Whereas no such treatment exists yet that can reverse these neurological conditions, treatments can help manage the symptoms. The treatment of dementia depends on the underlying causes and types of conditions. Lately, research has been mainly focused on small-molecule treatment approaches and immunotherapies. Still, the failure of tau-targeted and amyloid-targeted therapies should lead to researchers’ focus turning to alternative therapeutic strategies, such as stem cell-based therapy and gene therapy.

There is a dire need for planning, policymaking, and the allocating of resources to develop effective treatments and support the welfare of patients. A global effort can reduce this disease’s prevalence by improving treatment strategies and adopting preventive and disease-modifying interventions.

## Figures and Tables

**Table 1:** The list of current ongoing trials therapies based on mitochondrial complex I inhibitors and others.

Therapy Type	Drug Name	Under	FDA Status
Small Molecule	Metformin	Alzheimer’s Disease	AD (Phase 2/3)
Supplement, Dietary, Small Molecule	Resveratrol	Alzheimer’s Disease Mild Cognitive Impairment	AD (Phase 3) MCI (Phase 4)
Supplement, Dietary	Epigallocatechin Gallate (EGCG)	Alzheimer’s Disease	AD (Phase 2/3)
Small Molecule	Dexpramipexole	Alzheimer’s Disease Amyotrophic Lateral Sclerosis	AD (Phase 2) ALS (Discontinued)
Small Molecule	Masitinib	Alzheimer’s Disease Amyotrophic Lateral Sclerosis	AD (Phase 3) ALS (Phase 3)
Passive Immunotherapy	TB006	Alzheimer’s Disease	AD (Phase 1)

**Table 2:** Current amyloid-beta drugs of interest for AD.

Therapy Type	Drug Name	FDA Status
Passive immunotherapy	Solanezumab	Phase 3
Passive immunotherapy	Gantenerumab	Phase 3
Passive immunotherapy	Lecanemab	Phase 3
Passive immunotherapy	Crenezumab	Phase 3
Passive immunotherapy	Donanemab	Phase 3
Active immunotherapy	UB-311	Phase 3
Active immunotherapy	ABvac 40	Phase 2
Combination, Small Molecule	ALZT-OP1	Phase 3
Small Molecule	GV-971	Alzheimer’s Disease (Inactive) Approved in China

**Table 3:** Current ongoing clinical trial therapeutics of tau.

Therapy Type	Name	Under	Status
Passive immunotherapies	RO7105705	Alzheimer’s Disease	Phase 2
Passive immunotherapies	JNJ63733657	Mild AD	Phase 2
Passive immunotherapies	UCB0107	Progressive Supranuclear Palsy, Alzheimer’s Disease	PSP (Phase 1), AD (Phase 2)
Passive immunotherapies	PNT001	Alzheimer’s Disease, Traumatic Brain Injury	AD (Phase 1), TBI (Phase 1)
Passive immunotherapies	E2814	Alzheimer’s Disease	Alzheimer’s Disease (Phase 1/2)
Passive immunotherapies	LUAF87908	Alzheimer’s Disease	Phase 1
Active Immunotherapies	AADvac-1	AD, Progressive Nonfluent Aphasia	AD (Phase 2), PNA (Phase 1)
Active Immunotherapies	ACI-35	AD	Phase 2
Tau Aggregation Inhibitors (TAI)	Methylene Blue (LMTM)	AD, Frontotemporal Dementia	AD (Phase 3), FTD (Phase 3)
Post-translational modification aimed at tau	Lithium	MCI (Phase 4),	(Phase 4)
	Sodium selenate	AD	(Phase 2)

**Table 4:** Summary of studies on menopausal hormonal therapy (MHT).

Study, Year	Outcome
Yu Jin Kim 2021	Sample size and subject: 1,411,215 women (45 years or older) with or without HT medication history. Findings: 381,306 women were associated with a remarkably reduced risk of dementia (NDDs), and the more significant duration of therapy was associated with a reduced risk of NDDs, including AD and dementia.
Yoo, 2020	Sample size and subject: 4,696, 633 postmenopausal women without dementia. Findings: 212,227 incident cases of dementia; the use of hormone replacement therapy and oral contraceptive independently reduced the dementia risk by 15% and 10%, respectively.
Han, 2020	Sample size: 13,110 Findings: TIB use was not remarkably associated with the risk of dementia. A total of 940 incident cases of dementia, with 883 cases of AD and 206 VD.
Paganini-Hil, 2020	Sample size and subject: 424 women without dementia Findings: 209 incident cases of dementia; no individual menopausal, reproductive, menstrual, or estrogen replacements were linked with the risk of incident dementia after age 90.

**Table 5:** Synaptic therapeutics of interest.

Drug Name	Therapy Type	Condition	Status
AZD0530	Small molecule therapy	Alzheimer’s Disease, Parkinson’s Disease	AD (Discontinued), PD (Phase 1)
CT1812 (Elayta)	Small molecule therapy	Alzheimer’s disease	(Phase 2)

## Abbreviations

AAV—adeno-associated virus; ABP—androgen-binding protein; ABP—Aβ plaques; AD—Alzheimer’s disease; APP—amyloid precursor protein; ATP—adenosine triphosphate; Aβ—amyloid-p; BBB—blood–brain barrier; BDNF—brain-derived neurotrophic factor; CEE—conjugated equine estrogen; CSF—cerebrospinal fluid; DNA—deoxyribonucleic acid; ECM—extra cellular matrix; FDA—Food and Drug Administration; FDG-PET—fluorodeoxyglucose-positron emission tomography; HRT—hormone replacement therapy; LBD—Lewy body dementia; MHT—menopausal hormonal therapy; miRNA—micro ribonucleic acid; mtDNA—mitochondrial deoxyribonucleic acid; PD—Parkinson’s disease; PNS—peripheral nervous system; PTEM—platelet transmission electron microscope; RNA—ribonucleic acid; ROS—reactive oxygen species; SMR—standardized mortality ratio; U.S.—United States; WD—Western diet; WNT—wingless and Int-1.
